# Lack of an association between gallstone disease and bilirubin levels with risk of colorectal cancer: a Mendelian randomisation analysis

**DOI:** 10.1038/s41416-020-01211-x

**Published:** 2021-01-07

**Authors:** Richard Culliford, Alex J. Cornish, Philip J. Law, Susan M. Farrington, Kimmo Palin, Mark A. Jenkins, Graham Casey, Michael Hoffmeister, Hermann Brenner, Jenny Chang-Claude, Iva Kirac, Tim Maughan, Stefanie Brezina, Andrea Gsur, Jeremy P. Cheadle, Lauri A. Aaltonen, Malcom G. Dunlop, Richard S. Houlston

**Affiliations:** 1 Division of Genetics and Epidemiology, The Institute of Cancer Research, London, UK; 2 Cancer Research UK Edinburgh Centre and Medical Research Council Human Genetics Unit, Institute of Genetics and Molecular Medicine, University of Edinburgh, Edinburgh, UK; 3 Medicum and Genome-Scale Biology Research Program, Research Programs Units, Department of Medical and Clinical Genetics, University of Helsinki, Helsinki, Finland; 4 Centre for Epidemiology and Biostatistics, University of Melbourne, Melbourne, VIC Australia; 5 Centre for Public Health Genomics, University of Virginia, Virginia, VA USA; 6 Division of Clinical Epidemiology and Aging Research, German Cancer Research Center, Heidelberg, Germany; 7 Division of Preventive Oncology, German Cancer Research Center, Heidelberg, Germany; 8 German Cancer Consortium, German Cancer Research Center, Heidelberg, Germany; 9 Unit of Genetic Epidemiology, German Cancer Research Center, Heidelberg, Germany; 10 Cancer Epidemiology Group, University Medical Center Hamburg-Eppendorf, University Cancer Center Hamburg, Hamburg, Germany; 11 Department of Surgical Oncology, University Hospital for Tumours, Sestre milosrdnice University Hospital Centre, Zagreb, Croatia; 12 Department of Oncology, University of Oxford, Oxford, UK; 13 Institute of Cancer Research, Department of Medicine I, Medical University of Vienna, Vienna, Austria; 14 Institute of Medical Genetics, School of Medicine, Cardiff University, Cardiff, UK

**Keywords:** Colorectal cancer, Computational biology and bioinformatics

## Abstract

**Background:**

Epidemiological studies of the relationship between gallstone disease and circulating levels of bilirubin with risk of developing colorectal cancer (CRC) have been inconsistent. To address possible confounding and reverse causation, we examine the relationship between these potential risk factors and CRC using Mendelian randomisation (MR).

**Methods:**

We used two-sample MR to examine the relationship between genetic liability to gallstone disease and circulating levels of bilirubin with CRC in 26,397 patients and 41,481 controls. We calculated the odds ratio per genetically predicted SD unit increase in log bilirubin levels (OR_SD_) for CRC and tested for a non-zero causal effect of gallstones on CRC. Sensitivity analysis was applied to identify violations of estimator assumptions.

**Results:**

No association between either gallstone disease (*P* value = 0.60) or circulating levels of bilirubin (OR_SD_ = 1.00, 95% confidence interval (CI) = 0.96–1.03, *P* value = 0.90) with CRC was shown.

**Conclusions:**

Despite the large scale of this study, we found no evidence for a causal relationship between either circulating levels of bilirubin or gallstone disease with risk of developing CRC. While the magnitude of effect suggested by some observational studies can confidently be excluded, we cannot exclude the possibility of smaller effect sizes and non-linear relationships.

## Background

Gallstone disease has been proposed to increase the risk of colorectal cancer (CRC); however, evidence for a causal relationship from epidemiological studies is lacking.^1,2^ The observational nature of studies has made them vulnerable to confounding, from measured and unmeasured risk factors and reverse causation. It is especially noteworthy that many of the risk factors for gallstone disease include factors that are well-established as risk factors for CRC (obesity, high energy intake, alcohol consumption and diabetes).^3,4^ Increased production of bilirubin, the metabolic by-product of haemoglobin degradation, is also associated with gallstone disease.^5–10^ This is intriguing as bilirubin has anti-oxidant and anti-inflammatory attributes, with experimental studies reporting mildly elevated levels being associated with decreased oxidative stress-related disease, including cancer.^11–13^

Mendelian randomisation (MR) is an analytic methodology in which germline genetic variants are used as proxies, or instrumental variables (IVs), for putative risk factors. We assume that these genetic variants follow Mendel’s laws of inheritance, where the variants are randomly assorted at conception and are not in linkage disequilibrium (LD) with one another. These genetic variants are not influenced by reverse causation. The variants can also provide unconfounded estimates of disease risk, provided they do not directly influence the disease without complete mediation of the risk factor and are not associated with confounders of the relationship between risk factor and disease.^14–19^ Confounding can, however, be introduced to MR by population stratification, assortative mating and dynastic effects.^20^

To address the shortcomings of conventional observational epidemiological studies, we investigated whether genetic liability to gallstone disease and circulating levels of bilirubin are causally associated with CRC using MR. Specifically, we used two-sample MR, in which genetic variants associated with relevant risk factors as instrumental variables were first identified from genome-wide association studies (GWAS). We then assessed the association between these instrumental variables and CRC in a large GWAS.

## Methods

### Procedures

The genetic instruments, i.e., single-nucleotide polymorphisms (SNPs) to be used as IVs, were identified from recent meta-analyses or the largest GWAS published to date. Details on each meta-analysis are provided in Supplementary Tables 1 and 2. Briefly, summary statistics for gallstone disease were obtained from a meta-analysis of UK and Icelandic biobank data^21,22^ comprising 27,174 cases and 736,838 controls;^23^ effect sizes were not adjusted for covariates. Summary statistics for log bilirubin levels were obtained from a meta-analysis of three GWAS comprising 9464 individuals of Europeans ancestry;^24^ SNP-exposure effect sizes were age- and sex-adjusted.

To examine the association of each genetic instrument with CRC risk, we made use of summary effect estimates and corresponding standard errors (SEs) from a recent meta-analysis of 15 GWAS by Law et al.^25,26^ After imputation, this meta-analysis related over 10 million genetic variants to CRC in individuals of European ancestry. One of the 15 GWAS was an analysis of UK BioBank data. Since the published gallstone disease meta-analysis included UK BioBank data, we recomputed CRC association statistics excluding UK BioBank data to avoid bias from sample overlap. The 14 studies provided data on 26,397 patients with CRC and 41,481 controls. Aside from principal component adjustment for residual population substructure, none of the contributing GWAS were adjusted for covariates (Supplementary Table 3). Ethical approval for this study was not required as all data came from summary statistics and no individual-level data were used.

**Fig. 1:**
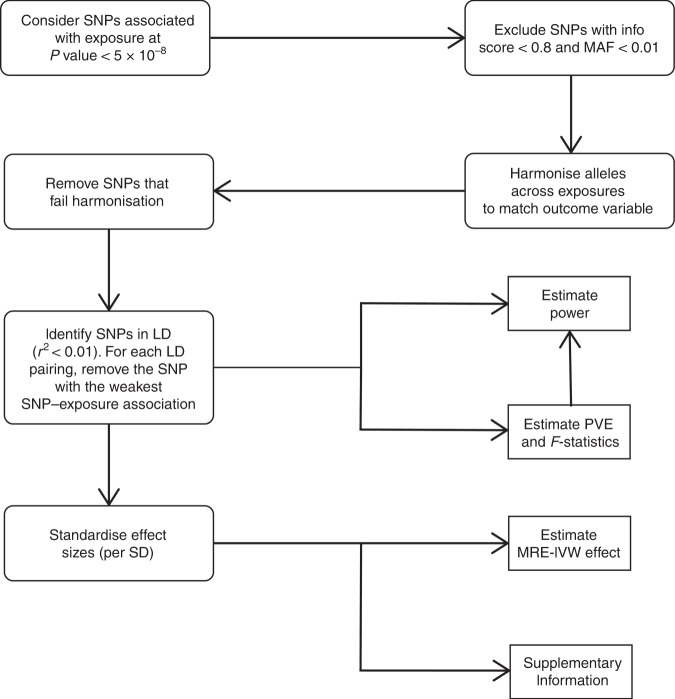
Flowchart of single-nucleotide polymorphism filtering. EA effect allele, MAF minor allele frequency, LD linkage disequilibrium, PVE, proportion of variation explained, SD standard deviation, MRE-IVW multiplicative random effect inverse variance weighted.

We only considered SNPs associated with each trait at *P* value of less than 5 × 10^−8^ with minor allele frequency greater than 0.01 in Europeans as potential instruments. We excluded SNPs with poor imputation quality (info score <0.8). For each SNP, the chromosome position was recovered, and effect estimates harmonised according to their effect alleles and corresponding frequencies in both datasets (failure in harmonisation, such as palindromic alleles, resulted in those SNPs being removed). Effect estimates were expressed in standard deviations (SD) of the trait per allele, along with the corresponding SE. While Ferkingstad et al.^23^ used a ‘multiplicative allele’ model in their analysis of gallstone disease, we assumed association estimates equated to those that would have been obtained using an additive model (an assumption our MR estimator applies). To avoid co-linearity between SNPs for each trait, correlated SNPs were excluded (LD threshold, *r*^2^ ≥ 0.01, with LD estimates for the European population obtained from MR-Base^17^) within each trait, with SNPs with the strongest effect size retained. Post filtering (Fig. 1), 26 and 2 SNPs were used as IVs for gallstone disease and circulating bilirubin, respectively (Supplementary Table 4).

**Fig. 2:**
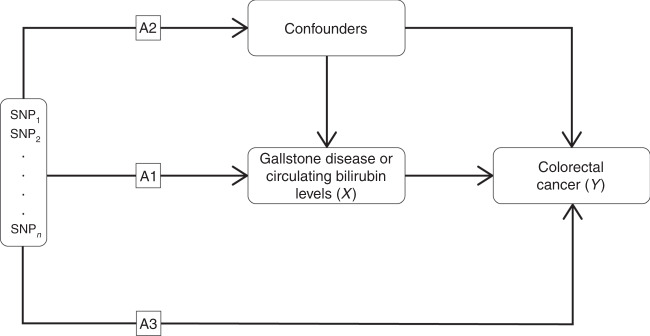
Mendelian randomisation (MR) and the key assumptions required to obtain an unbiased estimate of the causal effect. (A1) genetic variants used as instrumental variables are only associated with the modifiable risk factor (X, gallstone disease or circulating bilirubin levels); (A2) there exists no instrument-outcome confounding including, but not limited to, conventional confounders of the exposure-outcome relationship; (A3) genetic variants only influence the risk of CRC (Y) through the risk factor (X).

### Statistical analysis

The MR methodology assumes that genetic variants used as instruments for a risk factor are associated with only the risk factor and not with any confounders or another causal pathway (Fig. 2).^15–18,27,28^

To examine for a causal relationship between genetic liability to circulating levels of bilirubin and gallstone disease with CRC, we used a multiplicative random effect inverse variance weighted estimator (MRE-IVW)^28,29^ as our primary measurement. We adopted this method as it is robust in the presence of pleiotropic effects, provided any heterogeneity is balanced or normally distributed (centred at zero) and that the instrument strength independent of direct effect (InSIDE) and no measurement error (NoME) assumption is met.^19,28^ The results for the relationship between the levels of circulating bilirubin and CRC are reported as odds ratios per genetically predicted standard deviation unit increase (OR_SD_) and 95% confidence intervals (CIs). Two-sample MR analysis of the association between a binary risk factor (i.e., gallstone disease) and a binary outcome (i.e., CRC) is problematic as estimates can be positively biased should there exist a true causal association, although bias becomes less of an issue as the true causal effect tends to unity.^30,31^ In view of this when testing the association between gallstone disease and CRC, we primarily consider whether there exists a significant non-zero effect, and only report ORs for completeness.^32,33^ We assessed evidence of heterogeneity by Cochran’s *Q*,^28,34,35^ *I*^2^ statistics^35,36^ and by examining funnel and forest plots of Wald ratio estimates for SNPs. To test the robustness of findings, we also derived weighted median^37^ and weighted mode^38^ MR estimators. Evidence of directional pleiotropy was assessed using MR-Egger regression.^39,40^ We also formally assessed the effect of SNP heterogeneity on the MRE-IVW estimate, by implementing MR-PRESSO outlier and distortion tests.^41^

A Bonferroni-corrected *P* value of 0.025 (i.e., 0.05/2 putative risk factors) was considered significant, with a 0.025 <*P* value < 0.05 being considered suggestive of a causal association. The power of our MR analysis to demonstrate causal effects was estimated considering the proportion of variance (PVE) in the risk factor explained by the genetic instruments,^42,43^ stipulating a *P* value of 0.05. We assumed that the PVE explained by all variants combined was approximately equal to the sum of the individual PVEs as per Shim et al..^44^ Since calculation of power for binary exposure and binary outcome in a two-sample setting is problematic, we did not estimate study power for gallstone disease. Bias from weak instruments, or violation of the strong variant–risk-factor association assumption, affecting bilirubin effect estimates, were analysed using the estimated total PVE, the mean *F* statistic and the Staiger–Stock rule.^45^ Analyses were conducted using R v3.4.0^46^ and the TwoSampleMR R-package.^17^

## Results

**Fig. 3:**
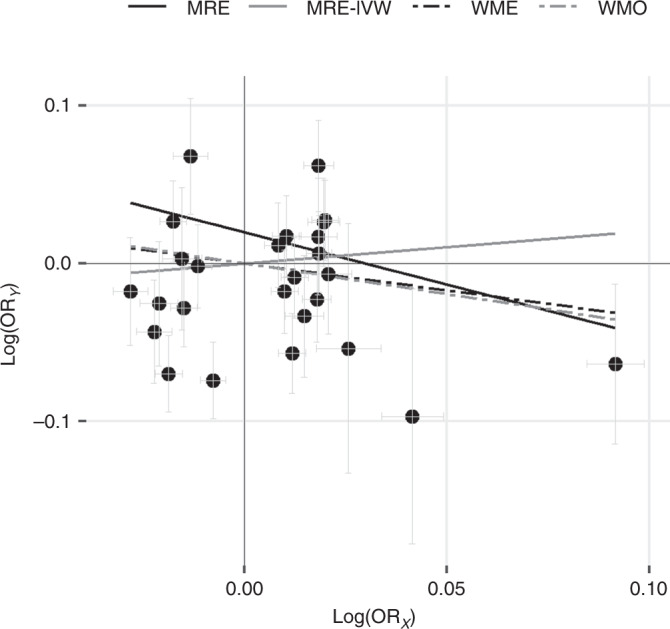
Single-nucleotide polymorphism (SNP) exposure association estimates for gallstone disease risk against the SNP-outcome association estimates for colorectal cancer risk. Causal effect given by each Mendelian randomisation (MR) estimators, caveated by issues discussed in the main text. MRE-IVW multiplicative random effects inverse variance weighted, MRE MR-Egger, WME weighted median, WMO weighted mode, log(OR_X_) log odds ratio (OR) per genetically predicted standard deviation increase in the exposure for each allele, log(OR_Y_) log odds ratio in the outcome for each additional allele.

**Fig. 4:**
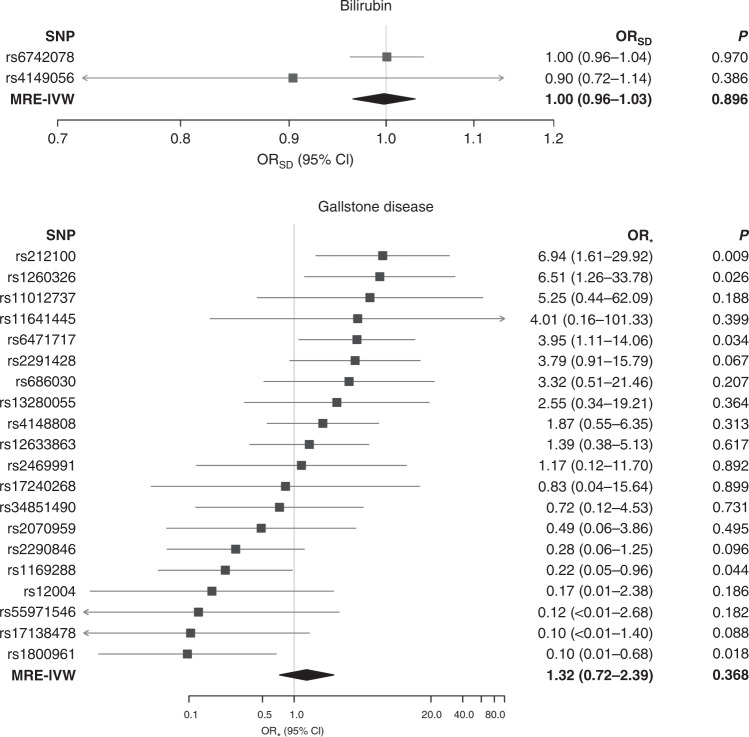
Forest plots of instrumental variable Wald ratios and causal effect estimates of the relationship between circulating bilirubin levels and gallstone disease with colorectal cancer. Causal effects estimated using the multiplicative random effects inverse variance weighted (MRE-IVW) method. OR_SD_ odds ratio per genetically predicted standard deviation unit increase in bilirubin, OR_*_ odds ratio per genetically predicted standard deviation (of the log odds of gallstone disease) log unit increase of the risk of gallstone disease, caveated by issues discussed within the main text; SNP single-nucleotide polymorphism, CI confidence interval.

Analysis on the basis of MRE-IVW provided no evidence to support a causal association between gallstone disease and CRC (*P* = 0.60). MR-Egger showed no evidence to suggest directional pleiotropy (*P* = 0.20). Following on from MRE-IVW, we examined the relationship between gallstone disease and CRC under weighted median, weighted mode and MR-Egger regression methodologies, all providing little evidence to support a causal association (*P* values of 0.23, 0.19 and 0.4, respectively, Table 1, Supplementary Tables 5–6, Figs. 3–4, Supplementary Fig. 1). There was evidence of heterogeneity between the SNPs used as IVs for gallstone disease (Cochran’s *Q P*  = 6.57 × 10^−23^, *I*^2^ = 85%), and the outlier test from MR-PRESSO identified six SNPs being responsible for this heterogeneity (Supplementary Table 4). Effect re-estimation excluding the outlying SNPs showed no evidence to support a causal association (*P* = 0.37). There was also no evidence to indicate that outliers were causing a large distortion in the causal association estimate (*P* = 0.77, Supplementary Tables 5–6, Fig. 4 and Supplementary Fig. 2).

**Table 1:** Two-sample Mendelian randomisation analysis of the relationship between circulating levels of bilirubin and gallstone disease with risk of colorectal cancer.

	OR_SD_	CI (95%)	*P* value
*Bilirubin*			
MRE-IVW	1.00	(0.96, 1.03)	0.90
*Gallstone disease*	OR_*_		
MRE-IVW	1.23	(0.57, 2.66)	0.60
WME	0.71	(0.41, 1.24)	0.23
WMO	0.68	(0.39, 1.19)	0.19
MR-Egger	0.52	(0.12, 2.27)	0.4

MRE-IVW multiplicative random effects inverse variance weighted, WME weighted median, WMO weighted mode, CI (95%) 95% confidence interval, OR_SD_ odds ratio per genetically predicted standard deviation unit increase in bilirubin, OR_*_ odds ratio per genetically predicted standard deviation (of the log odds of gallstone disease) log unit increase of the risk of gallstone disease, caveated by issues discussed in the main text.

The mean *F* statistic for the IVs used for genetically determined circulating level of bilirubin was 1038, corresponding to a PVE of 18%. Hence, the risk of weak instrument bias and violation of the NOME assumption was low. Specifically, we had 80% power to detect an OR_SD_ of 1.05 and over 90% power to detect an OR_SD_ of 1.10 for CRC (Supplementary Tables 4 and 5). Nevertheless, there was no evidence for a causal association between levels of circulating bilirubin and CRC risk using MRE-IVW methodology (OR_SD_ = 1.00, 95% CI = 0.96–1.03, *P* = 0.90, Table 1, Fig. 4). As only two IVs were used, other estimators such as MR-Egger could not be applied.

## Discussion

There has been interest in exploring a possible relationship between gallstone disease and risk of CRC since an association could be reflective of pathways linking bilirubin/bile acid metabolism, sex hormones and cholesterol metabolism. Previous epidemiological evidence for an association between gallstone disease and CRC has been inconclusive. Chen et al. reported that individuals with cholelithiasis had a higher risk of developing CRC (adjusted hazards ratio (AHR) = 1.36, 95% CI = 1.12–1.66). The risk increased for patients with cholelithiasis who went on to have a cholecystectomy (AHR = 1.56, 95% CI = 1.12–2.17).^1^ In comparison, Ward et al. concluded that the incidence of gallstone disease did not increase the risk, hazard ratios (HRs) were stratified by sex, of CRC (HR_Men_ = 0.81, 95% CI = 0.63–1.04; HR_Women_ = 1.14, 95% CI = 0.99–1.31).^2^

These epidemiological studies, however, differ substantially in design, sample size and their ability to adjust for key covariates. The large cohort studies with high numbers of gallstone cases having very limited covariate data and being heavily reliant on self-reported information. For example, in the EPIC cohort study, data on gallstones were missing from 17% of participants and cholecystectomy status was unknown.^2^ Moreover, the analysis was based solely on baseline data. Studies with more extensive data on potential confounders have typically been small, being based on fewer gallstone cases.^47^

An important strength of our analysis is that by utilising the random allocation of genetic variants, we were able to overcome potential confounding, for example, from other interrelated traits. Furthermore, reverse causation and selection bias may have biased estimates from previously published observational studies. Exploiting data from large genetic consortia for circulating bilirubin levels, gallstone disease and CRC risk has enabled us to more precisely test study hypotheses than if we had been reliant on individual-level data from a small study. The use of summary test statistics in two-sample MR analyses does, however, require consideration of sample overlap, the winner’s curse and genotype uncertainty. We have sought to avoid sample overlap between the association studies of the exposure traits and outcome trait by excluding CRC data based on analysis of UK BioBank.

In our MR, we have used genetic variants as proxies for circulating log levels of bilirubin and gallstone disease. Our findings suggest that neither trait is a major influence on the development of CRC. Our study has several advantages over those conducted previously. Firstly, we made use of summary GWAS data from a large published study of CRC. Secondly, provided the underlying assumptions of the MR estimator we use are met, the common sources of probable bias in previous conventional observational epidemiological studies, including reverse causation and residual confounding, will have been avoided, albeit with limitations and interpretation issues discussed below.

Firstly, as is the case with many MR analyses, despite our study being based on a large case–control data, we were not powered to detect small effects. For gallstone disease, we were unable to assess IV strength and power to detect causal effects as we are not aware of suitable methods for doing so when considering a binary exposure in a two-sample MR investigation. Moreover, we were not able to stratify by subgroups of interest, including sex and cancer site. This may be especially relevant, as some epidemiological studies have only reported significant associations for colon cancer in women. This lack of stratification or adjustment may also have led to bias in the causal estimates^18,48^ for bilirubin, as SNP–CRC association estimates were not adjusted for by age and sex. For bilirubin, we were not able to model non-linear associations as this would have required us to have access to individual data.

Analysing a binary exposure and a binary outcome in a two-sample MR setting is problematic with Wald-type ratio estimates potentially being positively biased should there exist a true causal association between a binary exposure and a binary outcome.^30,31^ Simulations performed by Didelez et al. demonstrated that if standard IV assumptions were met, one-sample IV estimators do not misspecify a no-association effect as being false. Disney-Hogg et al.^31^ arrived at the same conclusion using two-sample IVW estimators. Both simulation studies did however report inflated causal effect magnitudes when causal associations were simulated. Based on such considerations, we believe that our gallstone-CRC causal effect estimates, and the corresponding *P* values, will only have been subjected to minimal bias. Nevertheless, in analysing the relationship between gallstone disease and CRC, we focused on testing whether there exists a causal association and not principally on the magnitude of any effect.^32,33^

In conclusion, our findings shed light on an area for which the evidence to date has been mixed. Specifically, they provide evidence against the levels of circulating bilirubin or gallstone disease as major risk factors for CRC development.

## Supplementary information

Supplementary Figures, Legends and Info

Supplementary Tables

## Data Availability

Genetic instruments can be obtained through MR-Base or from published work.^23,24^ Details and availability of CRC genotyping data that support the findings of this study have been previously published.^26^
