# Supplementary material for: Lack of an association between gallstone disease and bilirubin levels with risk of colorectal cancer: a Mendelian randomisation analysis
Source: Br J Cancer. 2021 Jan 7;124(6):1169–74. doi: 10.1038/s41416-020-01211-x (PMC7961009; doi:10.1038/s41416-020-01211-x)
Supplement: Supplementary file 1 — Supplementary Figures, Legends and Info [file 41416_2020_1211_MOESM1_ESM.docx]

**
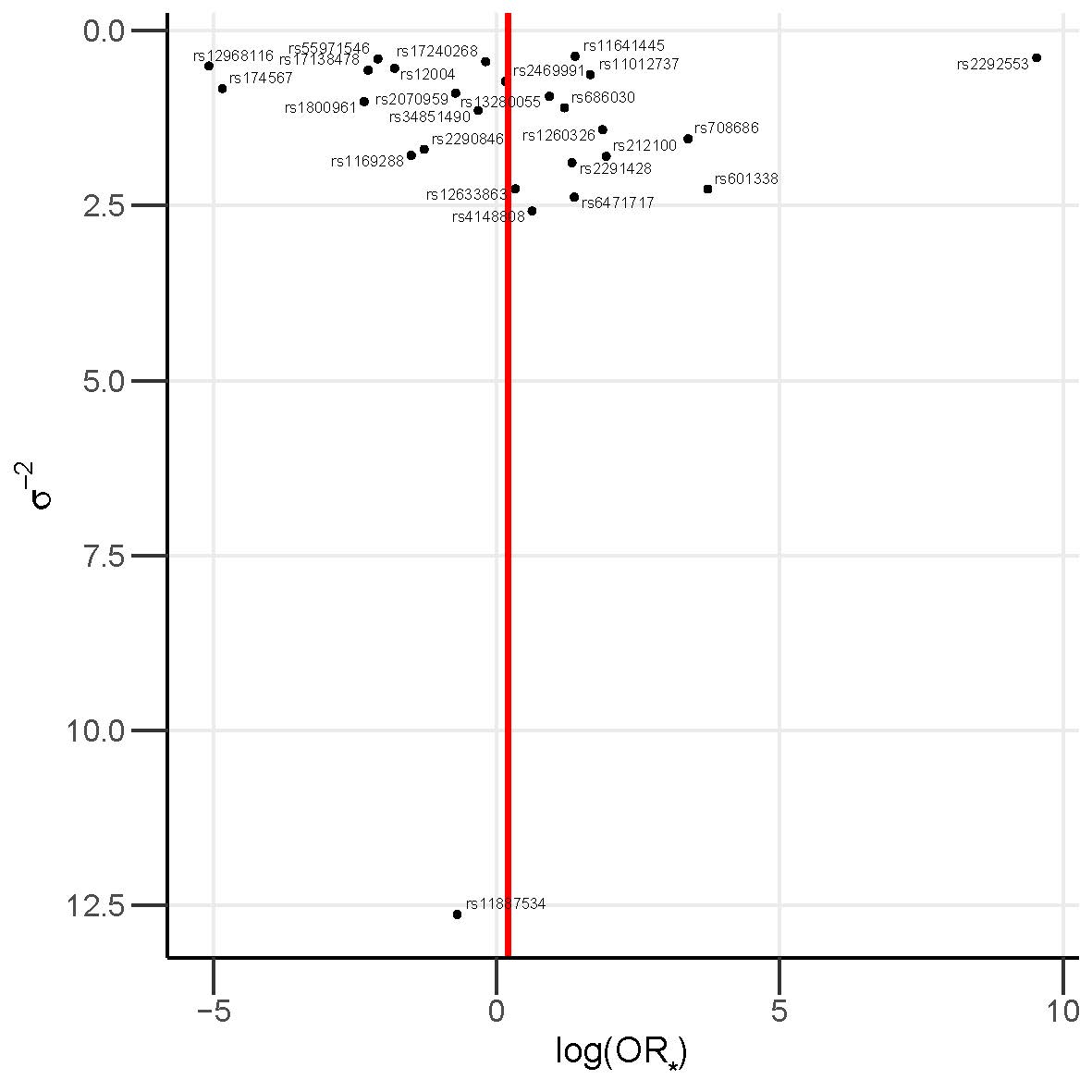
**

**Supplementary Figure 1: Funnel plot of causal effect estimates for gallstone disease on colorectal cancer risk, with all 26 valid instrumental variables.** Red line shows multiplicative random effects inverse variance weighted causal effect estimate. $\text{log}(\text{OR}_{\boldsymbol{*}})$, log odds ratio per genetically predicted standard deviation (of the log odds of gallstone disease) log unit increase of the risk of gallstone disease; $\sigma^{-2}$, Wald ratio estimate precision.

**
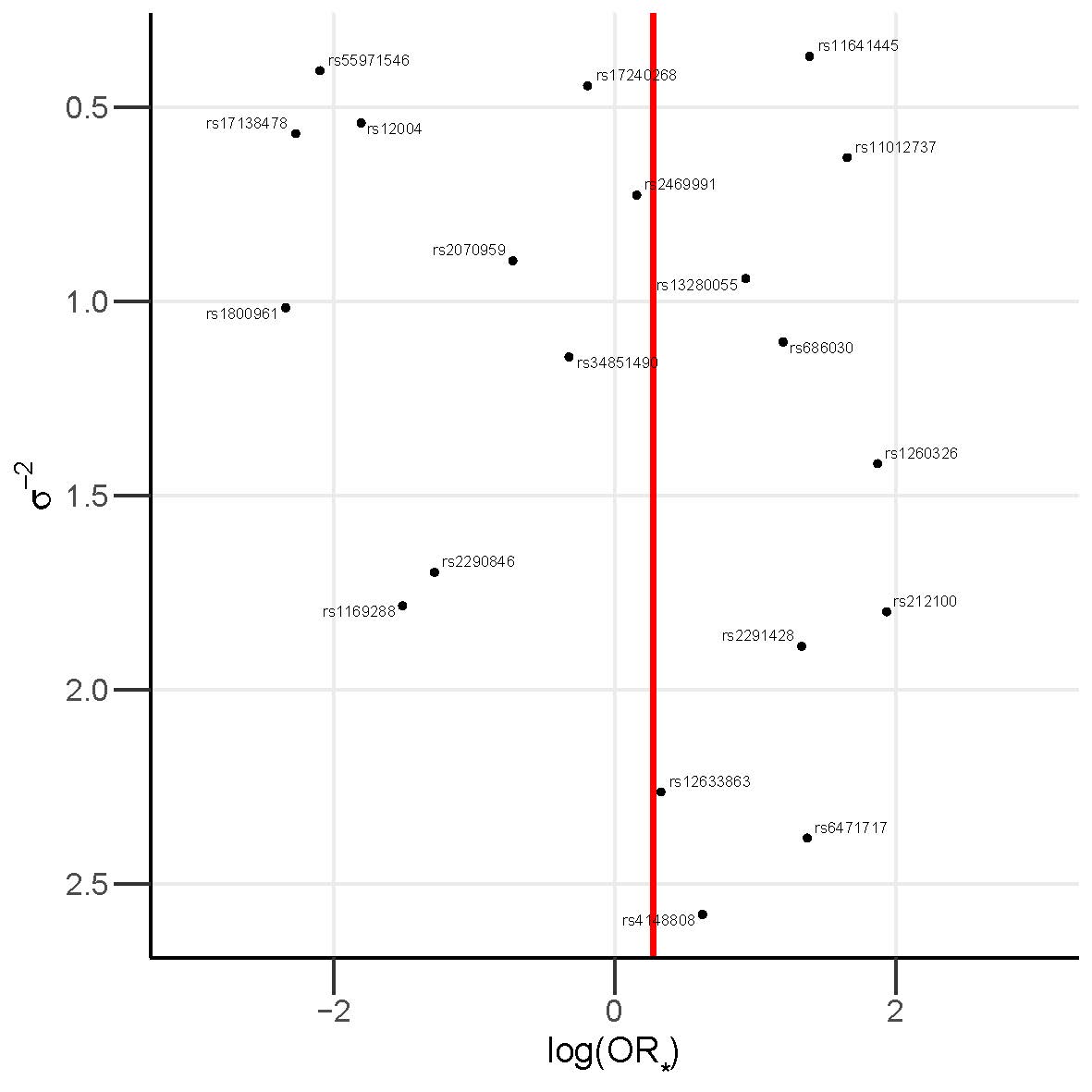
**

**Supplementary Figure 2: Funnel plot of causal effect estimates for gallstone disease on colorectal cancer risk excluding six single nucleotide polymorphisms showing heterogeneity excluded.** Red line shows multiplicative random effects inverse variance weighted causal effect estimate. $\text{log}(\text{OR}_{\boldsymbol{*}})$, log odds ratio per genetically predicted standard deviation (of the log odds of gallstone disease) log unit increase of the risk of gallstone disease; $\sigma^{-2}$, Wald ratio estimate precision.

**Supplementary Tables Legends:**

**Supplementary Table 1:** Gallstone disease GWAS demographic summary.

**Supplementary Table 2:** Circulating bilirubin levels GWAS demographic summary.

**Supplementary Table 3:** Summary of the 14 CRC GWAS in the meta-analysis.

**Supplementary Table 4:** Single nucleotide polymorphisms (SNPs) used as instrumental variables in the Mendelian randomisation analysis.

**Supplementary Table 5:** Assessing the suitability of IVs for each exposure for use in Mendelian randomisation analysis.

**Supplementary Table 6:** Causal estimates from each Mendelian randomisation method for each exposure and CRC risk.

**Supplementary Figure Legends:**

**Supplementary Figure 1: Funnel plot of causal effect estimates for gallstone disease on colorectal cancer risk, with all 26 valid instrumental variables.** Red line shows multiplicative random effects inverse variance weighted causal effect estimate. $\text{log}(\text{OR}_{\boldsymbol{*}})$, log odds ratio per genetically predicted standard deviation (of the log odds of gallstone disease) log unit increase of the risk of gallstone disease; $\sigma^{-2}$, Wald ratio estimate precision.

**Supplementary Figure 2: Funnel plot of causal effect estimates for gallstone disease on colorectal cancer risk excluding six single nucleotide polymorphisms showing heterogeneity excluded.** Red line shows multiplicative random effects inverse variance weighted causal effect estimate. $\text{log}(\text{OR}_{\boldsymbol{*}})$, log odds ratio per genetically predicted standard deviation (of the log odds of gallstone disease) log unit increase of the risk of gallstone disease; $\sigma^{-2}$, Wald ratio estimate precision.
